# Rosiglitazone but not losartan prevents Nrf-2 dependent CD36 gene expression up-regulation in an *in vivo *atherosclerosis model

**DOI:** 10.1186/1475-2840-7-3

**Published:** 2008-02-26

**Authors:** Y Hernandez-Trujillo, F Rodriguez-Esparragon, A Macias-Reyes, A Caballero-Hidalgo, Jose C Rodriguez-Perez

**Affiliations:** 1 Research Unit, Hospital Universitario de Gran Canaria Dr. Negrín, Las Palmas de Gran Canaria, Spain; 2 Nephrology Service, Hospital Universitario de Gran Canaria Dr. Negrí, Las Palmas de Gran Canaria; 3 Deparment of Medical and Surgical Sciences, Universidad de Las Palmas de Gran Canaria (ULPGC), Las Palmas de Gran Canaria. Spain

## Abstract

**Background:**

Thiazolidinediones exert anti-inflammatory and anti-oxidative roles and attenuate atherosclerosis by mechanisms partially independent of their metabolizing actions. High doses of angiotensin type 1 receptor (AT_1_R) blocker losartan (LST) seem to promote fat cell formation by preserving PPARγ activity.

**Methods:**

C57BL/6J diet-induced atherosclerotic susceptible mice randomly received a normal or a high-fat high-cholesterol (HFHC) diet and were treated with rosiglitazone (RG), LST or a vehicle for 12 weeks.

**Results:**

HFHC was associated with increased PPARγ gene expression without an over regulation of PPARγ responsive genes, whereas RG and LST treatments were found to maintain PPARγ activity without resulting in increased PPARγ gene expression. A better anti-inflammatory and antioxidant profile in mice treated with RG regarding LST was observed in spite of a similar PPARγ preserved activity. Chromatin immunoprecipitation (ChIP) assays revealed that animals under HFHC diet treated with RG showed a significant nuclear factor erythroid 2-like 2 (Nrf2)-dependent down-regulation of the expression of the CD36 gene.

**Conclusion:**

The PPARγ agonist RG exerts antioxidant properties that significantly reduced Nrf-2-dependent CD-36 up-regulation in mice under HFHC diet. Because LST treatment was also associated with a preserved PPARγ activity, our data suggests that these RG antioxidant effects are partially independent of its PPARγ metabolizing properties.

## Background

Superoxide generation occurs in conditions such as hypertension, hypercholesterolemia, diabetes, and cigarette smoking. Oxidant stress alters many functions of the endothelium which, associated with traditional risk factors, triggers early vascular inflammation and a predisposition to atherosclerosis [1-3]. Low-density lipoproteins (LDLs) are susceptible to oxidative damage. Oxidative stress-mediated LDLs modification (ox-LDLs) has a key role in initiation of the atherosclerotic process [1,4,5]. Ox-LDLs are taken up via different scavenger receptors. The CD36 scavenger receptor constitutes the major ox-LDL receptor [6]. Studies employing transgenic and knock-out mice have demonstrated that CD36 is proatherogenic according to observations that targeted disruption of the gene was protective against atherosclerosis [7]. Components of ox-LDL activate peroxisome proliferator-activated receptor γ (PPARγ) resulted in an up-regulation of the CD36 scavenger receptor [8,9].

Thiazolidinediones (TZDs) are PPARγ agonists that improve insulin sensitivity, reduce triglyceride levels and decrease the risk of atherosclerosis in diabetic patients. TZDs also exert direct effects on vascular wall cells [10-12]. TZDs treatments inhibit intimal lesions [13], suppress monocyte elaboration of inflammatory cytokines [14], macrophage activation [15], and the expression of cell adhesion molecules [16]. TZDs also increase the mRNA expression of the proatherogenic CD36 gene. PPARγ seems to be determinant in the CD36 gene regulation as deduced from macrophages derived from mice in which the PPARγ gene has been "floxed out" [17]. However, most authors suggest that the CD36-increasing effects of TZDs might be overwhelmed by the antiatherogenic effects of other factors. Rosiglitazone (RG) is a TZD that possesses several anti-inflammatory properties demonstrating a protective action in regulating atherosclerosis development [18-20].

Nuclear factor-erythroid 2-related factor 2 (Nrf2) is a member of the Cap 'n' Collar family of basic region-leucine zipper transcription factors. Transcription factor Nrf2 is a major regulator of genes encoding phase 2 detoxifying enzymes and antioxidant stress protein in response to electrophilic agents and oxidative stress. Evidence that ox-LDLs and some lipid hydroperoxides contained in ox-LDL enhanced nuclear levels of Nrf2 in macrophages has been reported [21]. This increase results in an up-regulation of the scavenger receptor CD36 and antioxidant stress proteins [21]. Thus, Nrf2 appears as an additional transcription factor regulating CD36 gene expression.

Adipocyte differentiation seems to be linked to an improvement of insulin sensitivity [22]. Sharma et al [23-25] proposed the hypothesis that blockade of the renin-angiotensin system prevents diabetes by promoting the recruitment and differentiation of adipocytes. *In vitro *studies further demonstrate that several angiotensin receptor blockers or their metabolites induce PPARγ activity and opposite, ligand activated-PPARγ suppressed AT_1_R gene transcription [25-27]. Paradoxically, studies reveal that a moderate reduction of PPARγ activity as observed in heterozygous PPARγ-deficient mice, or the Pro12Ala polymorphism in human PPARγ gene, prevent insulin resistance and obesity induced by a high-fat diet [28]. This reduction is thought to occur because PPARγ expression is regulated by nutritional state [29]. Oxidative and inflammatory mediators as those produced in long-term high-fat high-cholesterol (HFHC) diets are known to be inducers of different responses that regulate PPARγ gene activity. The activity of PPARγ can be finely tuned through integration of diverse phosphorylation events [29-32].

The aim of the present study was to show that RG and losartan (LST) treatments preserved the liver PPARγ activity in mice that were fed with a HFHC diet. On the contrary, a loss of PPARγ activity was observed instead of an increase of the PPARγ gene expression in the untreated hypercholesterolemic control mice. Lipid hydroperoxides levels and the relative expression values of the inflammatory mediators inducible nitric oxide synthase (iNOS) and Interleukin-6 (IL-6) genes were diminished in mice treated with RG with respect to LST treated mice. These findings were suggestive of RG additional properties independent of its metabolizing actions. A difference in the CD36 gene expression level between treated groups was observed. Because oxidative stress activates Nrf2 responses and recently has been described as the essential contribution of Nrf2 to the CD36 gene expression, we also investigated whether Nrf2 mediates the CD36 gene up-regulation observed in the LST group. A chromatin immunoprecipitation (ChIP) assay revealed that in the RG prevents Nrf2-dependent CD36 up-regulation.

## Methods

### Animals

Two-month old female inbred strain C57BL/6J and C3HeJ/6J mice that differ strikingly in aortic fatty streak development when fed a high-fat diet were housed at 25°C on a 12-hour light/dark cycle and provided with food and water *ad libitum*. At day 0, C57BL/6J animals were maintained either on a chow diet or a high-cholesterol diet containing 15% fat, 1.25% cholesterol, and 0.5% cholic acid (Panlab, S.L., Barcelona, Spain) for 12 weeks. Twenty-three mice per group were randomly selected in one of the following groups: group 1 (control) was maintained on chow diet; group 2 (control) was maintained on high-cholesterol diet; group 3 constituted C57BL/6J mice maintained on high-cholesterol diet that received RG administered at 25 mg/Kg/day; and group 4 was composed of C57BL/6J mice on high-cholesterol diet that received LST administered in the drinking water at 100 mg/L. RG was administered by food and was monitored every day. Similarly, the water consumption in the LST group was monitored weekly. Each mouse was estimated to drink 1.5 ml of water/10 grams of body weight/day. A group of 25 C3HeJ female mice fed on high-cholesterol diet was also created (group 0).

All experimental procedures were performed in accordance with the animal welfare guidelines of the Hospital Universitario de Gran Canaria Dr. Negrín; the national guide (Royal Decree 223/1988) and the international guide (Guide for the Care and Use of Laboratory Animals, published by the US National Institutes of Health) for the use and care of animals for experimentation. All investigators directly involved in the handling of animals possess the relevant approvals from the Ministry of Agriculture, Fisheries and Food of the Directorate General of Animal Husbandry of the Canary Islands Government in the B and C categories (Royal Decree 1201/2005).

Animals were anesthetized with 5 μl per grams of body weight using an anesthetic cocktail of Ketolar (ketamine hydrochloride at 50 mg/ml) (Parke-Davis, S.L.; Barcelona, Spain) and Rompún (xylazine at 2% administered at 2.5 mg/ml; Bayer Health Care, AG, Germany) in saline solution. Blood samples were collected by cardiac left ventricular punction in three animals per group, chosen between housed at days 1, 25, 50, 75, and 100. At the end of the study period (day 105) five mice per group were anesthetized and transcardially perfused with saline following tissue samples collection. Liver samples were obtained, snap-frozen by immersion in liquid nitrogen and stored at -20°C until use.

### Analysis of plasma lipids and lipid peroxidation

Blood was collected from 6-hour fasted animals and plasma samples were stored at -70°C. Insulin was measured by the Abbott kit insulin (Abbott Laboratories, USA). Plasma levels of total cholesterol (TC), triglyceride (TG) and HDL-cholesterol were measured enzymatically by using Wako kits. Total plasma lipid hydroperoxide (LOOH) content was calculated by the Xylenol Orange (FOX) assay as described elsewhere [33] and analyzed after being mixed with copper sulphate at a final concentration of 10 μM at 37°C for 24 h, using triphenyl phosphine (TPP) for signal authentication. The absorbance of the supernatants was monitored at 560 nm and the hydroperoxide content determined using a molar absorption coefficient of 4.3 × 10^4 ^M^-1^.cm^-1 ^or by reference to an H_2_O_2 _standard curve.

LOOH content was also measured in the supernatant of cultured cells by the modified method of Auerbach [34]. To 40 μl aliquots were added to a 96-well microtiter plate containing ethanol (10 μL) and *N*-benzoyl leucomethylene blue (LMB) color reagent (100 μL). The LMB reagent was prepared with 5 mg of LMB (Tokyo Kasei Kogyo) dissolved in 8 mL of *N*,*N*-dimethyl-formamide and 90 mL of 0.05 mol/L potassium phosphate buffer, pH 5, 1.4 g of Triton X-100, and 5.5 mg of hemoglobin. Linoleic acid hydroperoxide (13-hydroperoxy-9,11-octadecadienoic acid, ranging from 1 to 20 nmol in 10 μL ethanol) was added as a standard to wells containing saline (40 μL) and LMB. After 40 minutes at room temperature, the standards and samples were read at 650 nm using a microtiter plate reader. A standard curve for absorbency versus LOOH concentration was generated, and the hydroperoxide levels in samples were determined from this curve.

### Analysis of Paraoxonase/arylesterase activities

Paraoxonase (PON p. ase) and arylesterase (PON ar.ase) activities were evaluated by previously described methods [35,36].

### Analysis of Total Nitric Oxide Metabolites

Plasma samples were filtered through Microcon YM-10 columns (Millipore). Total nitric oxide metabolites concentration was evaluated in sample columns effluent by using a colorimetric kit assay according to the manufacturer instructions (R & D Systems).

### Cell-Free Dichlorofluorescein (DCF) assay

Total HDL was prepared by precipitation using the DS1 reagent. DS1 was prepared dissolving in water 10 mg/L dextran sulphate (Dextralip^®^50, Sigma), 0.5 mol/L MgCl_2_, and 0.05% sodium azide. The protein content of precipitated HDL was determined and HDL samples were subsequently adjusted to similar protein content with saline.

The cell-free Dichlorofluorescein (DCF) assay was performed essentially as described [37]. Ten microliters of DCFH-DA (Molecular Probes, Eugene, OR) dissolved methanol were placed in polypropylene tubes and evaporated under vacuum. A similar procedure was conducted following addition of L-α-1-palmitoyl-2-arachidonoyl-sn-glycero-3-phosphocholine (PAPC) (10 μl at 2.5 mg/ml in chloroform) and 20 μl of HPODE at 0.5 mg/ml in ethanol. Finally, 25 μl of saline or HDL at 500 μg/ml was added to each tube and the volume adjusted to 1 ml with saline. Samples were incubated for 2 hours at room temperature in the dark before counting in a FLx800 Microplate Fluorescence Reader. Authentic L-α-1-palmitoyl-2-arachidonoyl-*sn*-glycero-3-phosphocholine (PAPC) was obtained from Avanti Polar Lipids (Alabaster, AL) and 13(*S*)-HPODE was obtained from Biomol (Plymouth Meeting, PA).

### Cell Culture and Stimulation

3T3-L1 pre-adipocytes were grown in Dulbecco's modified Eagle medium (DMEM) to confluence and differentiated with 0.5 mM of isobuthylmethylxanthine (IBMX), 1 μM dexamethasone, 10 μg/ml insulin and 5% calf serum for 48 hours essentially as described [26]. Insulin was maintained for 2 additional days in the differentiated control cells group. For the remaining groups, cells were washed with PBS and incubated in medium containing RG at 40 μM, LST at 100 μM, or a vehicle for 5 days. Medium was replaced every day. At day 5, quiescent cells incubated with RG and LST were exposed to oxidized PAPC (ox-PAPC) for 24 h. Oxidized PAPC (ox-PAPC) was prepared and isolated as described [38]. 3T3-L1 preadipocyte and adipocytes were stained with Oil Red O essentially as described [39].

### Analysis of mRNA levels by real-time PCR

Total RNA was isolated from mice liver and cells by the Chomczynski method. Hepatic and cultured cells cDNAs was synthesized using MMLV (Roche Diagnostics) following the instructions provided by the manufacturer. Real-time PCR was performed on a LightCycler device using the FastStart DNA Master SYBR Green I (Roche Diagnostics) according to the provided protocol. Amplifications of PPARγ gene and of the PPARγ target gene SCARB1 were performed using the primers pairs: 5'-TTTTCAAGGGTGCCAGTTTC-3' and 5'-AATCCTTGGCCCTCTGAGAT-3' spanning 198 base pairs of the exon 6 of the PPARγ gene [GenBank accession number NM_011146] and 5'-GAAAAAGCGCCAAGTACAGC-3' and 5'-CAGGCTGTGGGAACTCTAGC-3' spanning 248 bp of the SCARB1 gene [GenBank NM_016741]. Fragments of the CD36; Paraoxonase 1 (PON1); 5-lipoxygenase (Alox5); 15 lipoxygenase type A (Alox15) and 15 lipoxygenase B (Alox15b) genes were amplified using the following primer pairs: 5'-GAGCAACTGGTGGATGGTTT-3' and 5'-GCAGAATCAAGGGAGAGCAC-3' which yields a 207 bp fragment of the CD36 gene [GenBank NM_007643]; 5'-CACCCGTCTCGATTCCTTTA-3' and 5'-CAGCCTGTCCATCTGTCTCA-3' that amplified a 178 bp fragment of the PON1 gene [GenBank NM_011134]; 5'-GTGCTGCTTGAGGATGTGAA-3' and 5'-CTACGATGTCACCGTGGATG-3' that amplified a 235 bp fragment of the Alox5 gene [GenBank L42198]; 5'-GATTGTGCCATCCTTCCAGT-3' and 5'-CAGGGATCGGAGTACACGTT-3' that amplified a 186 bp fragment of the Alox15 [GenBank NM_009660]; 5'-GCTAAAGCATGCCAGTGTGA-3' and 5'-GCTCTGATTAACGGCTTTGC-3' that yield a 229 bp fragment of the Alox15b gene [GenBank NM_009661]. Fragments of the IL-6, CYBA (p22phox), eNOS and iNOS genes were amplified using previously described primers [40,41]. When linearity and efficiencies were similar between targets and the reference amplified glyceraldehyde-3-phosphate dehydrogenase (GAPDH) fragment, relative expression analyses were performed by the comparative threshold cycle quantitation method. Alternatively, targets and the GAPDH fragment were analyzed by using serial dilutions of pooled cDNA and then applying the standard curve quantitation method. The 223 bp fragment of the GAPDH gene was amplified with the following primers: 5'-ACA CAT TGG GGG TAG GAA CA-3' and 5'-AAC TTT GGC ATT GTG GAA GG-3' [GenBank NM_008084]. Primers design was done by using the public domain software Primer3 [42].

### Chromatin immunoprecipitation assays

Liver samples (≈ 0.05 grams per mouse) were allowed to thaw on ice in serum-free DMEM and then cross-linked with 5% formaldehyde for 5 minutes. Liver fragments were homogenized and nuclear and cytosolic fractions extracts prepared by using a nuclear extraction kit from Active Motif according to the manufacturer instructions. Nuclear lysates were sonicated on ice. As a positive control (input) undiluted aliquots were retained for further processing in parallel with all the other samples at the reversal of the crosslinked step. ChIP assays were performed adding to the chromatin complexes 10 μl of αNrf2-C20 or PPARγ (H-100) antibodies (Santa Cruz) or without antibody overnight at 4°C. DNA samples were recovered and subjected to analysis by real time PCR amplification. Primers used were specifically designed to amplify a putative antioxidant response element (ARE) in the CD36 gene promoter and a putative peroxisome proliferator response element (PPRE) in the Apolipoprotein A-I (Apo A-I) gene promoter by using the software Primer3. Primers flanking a PPRE at iNOS gene were as described [43]. A putative CD36 ARE (RTGAYnnnGC) site was identified located between base positions 1425 to 1434 in the CD36 gene promoter [GenBank AF434766] and primers flanking this sequence were designed with the sequences: 5'-TGCTCTGAGCTCTACCCTCTG-3' and 5'-TCTGTCCCCTCTTTTGCAGT-3'. The CD36 promoter sequence was analyzed and completed for gap and errors considering the previously published information [44,45]. Mouse sequence data homologous to the Apo A-I 5' UTR region of the *Rattus norvegicus *Apo A-I promoter sequence [GenBank X54210] was identified after a BLAST search. A large fragment encompassing 562 bases from 171971 to 172532 of the *Mus musculus *chromosome 9 sequence, clone RP24-302M3 [GenBank AC116503], matched with the promoter sequence and was subsequently explored to find potential PPRE binding sites. A consensus sequence of the form RG(G/T)TCA was found. The identified sequence AGTTCAAGGATCA constitutes a DR1 element of the form with a perfect RG(G/T)TCA 5'-motif and an imperfect 3'-motif. Primers flanking a putative PPRE in the apo A-I promoter regions used were 5'-TCTGGGTGTCCAGCTCTTCT-3' and 5'-CCTGTTTGCCCACTCTGTTT-3'. Similarly, a BLAST search was performed using the SCARB1 promoter region of *Rattus norvegicus *[gi:50881992]. A fragment that was in common with the BAC clone of the *Mus musculus *chromosome 5 [gi:71533446] was selected. A PPRE site was identified in the fragment encompassing 360 base pair. This sequence was used to design primers flanking the SCARB1 promoter region with the sequences 5'-TTGTGTCCTGAGTGGAATGC-3' and 5'-AGCAGGGTGGTAGGGACTCT-3'. The CD36, SCARB1 and apo A-I promoter regions were also amplified by standard PCR and subjected to gel electrophoresis. Agarose bands were excised, purified (QIAGEN Gel Extraction Kit) and sequenced on an ABI Prism 310 device using the BigDye^® ^v. 3.1 Terminator Cycle Sequencing Kit according to the manufacturer instructions (Applied Biosystems).

### Statistical analyses

Quantitative variables are presented as mean ± SEM unless otherwise specified and qualitative variables as percentages. Pair wise related group comparisons were performed using the Wilcoxon test whereas the Mann-Whitney U-test was used for comparisons of unrelated groups. The level of significance was set at *P *< 0.05.

## Results

### Lipid profile and HDL concentration between treated groups

No differences in the food and water consumption were visible among animals of one experimental group or between experimental or control groups. There were no significant differences in insulin levels between treated and untreated mice.

**Figure 1:**
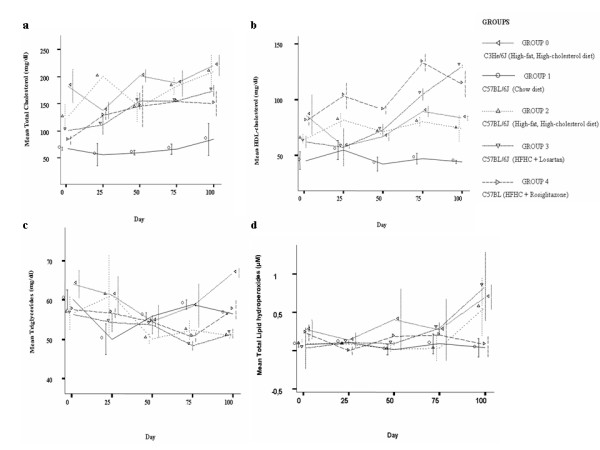
**Mean total cholesterol (**a**), HDL-cholesterol (**b**), triglycerides (**c**) and lipid hydroperoxides (**d**) content in plasma of study groups.** Triplicate measurements were taken in three animals per group before the onset of the study period (day 0), at day 1 and each 25 days elapsed after completing 100 days. Inter-group comparisons were made at day 1 and at day 100 and intra-group between days 1 and 100. Data are expressed as mean ± SD.

Animals that were fed on HFHC treated with RG showed a significant difference in HDL-cholesterol level with respect to mice fed only on HFHC diet at the beginning (p = 0.02) and at the end of the study period (p = 0.019). Mice treated with RG showed a significant increase in HDL-cholesterol levels between day 1 and day 100 (p = 0.016) (Figure 1b). No differences in total cholesterol and triglycerides levels were observed.

As shown in Figure 1d, we found lower, albeit not significant (p = 0.06), levels of total LOOH in mice fed on HFHC diet treated with RG with respect to those observed in mice fed on HFHC diet. Also, lower levels of LOOH were observed in the RG group with respect to mice treated with LST or even with respect to the C3HeJ control group. A significant difference in LOOH content in the RG group between days 1 and 100 (p = 0.02) was observed.

**Figure 2:**
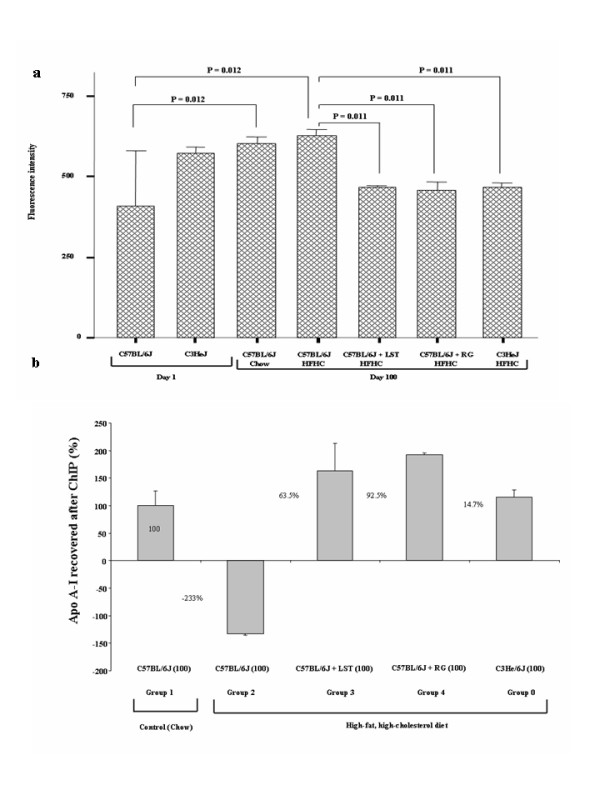
**Treatments preserve HDL antioxidant properties and apolipoprotein A-I-(apo A-I)-PPARγ-mediated gene expression.** (**a**). Fluorescence intensity (arbitrary units) was determined as described in Methods. There was no difference in the fluorescence signal levels measured of the HDL isolated from mice under control diet with respect to that obtained from mice that fed a HFHC diet after 1 day elapsed; thus the average fluorescence value obtained of the HDL from both groups was taken as "Day 1" reference value. Data are mean ± SD from thee separate experiments. (**b**). ChIP with a PPARγ antibody of study groups were used to amplify an apo A-I promoter fragment. ChIP figures percentages indicate increments or decrements regarding C57BL/6J mice fed HFHC diet. Data of C57BL/6J mice fed chow diet was established as 100%.

As pointed out above, raised HDL levels in RG with respect to LST treated mice were observed. Increase in apoA-I gene, a main determinant of HDL concentration levels, might occur after RG treatment because of its ability to act on PPRE elements located at the apoA-I gene which might also preserve HDL-associated antioxidant properties [18]. Therefore, these two possibilities were analyzed as follows. First, we tested the ability of isolated HDL to inhibit the oxidation of PACP by HPODE by the DCF cell-free assay. Fluorescence signal comparisons between HDL obtained from either C57BL/6J mice on a chow diet and C57BL/6J that fed a HFHC between day 1 and day 100 were significant (Figure 2a). Therefore, both time elapsed and hypercholesterolemic diet were associated with a reduction of the HDL antioxidant properties. As deduced from Figure 2a, we observed that both RG and LST treatment preserved the antioxidant properties of HDL. Interestingly, no differences in the HDL-linked Paraoxonase and arylesterase activities as well as in the PON1 gene expression were observed between pharmacological treated groups (not shown). Second, the Apo A-I proximal promoter levels were evaluated after PPARγ ChIP assay as described in our methods. As depicted in Figure 2b, experiments demonstrated respectively a 63.5% and a 92.5% of DNA amplified after ChIP in the LST and RG treated mice groups with respect to mice fed on HFHC diet. The difference between both pharmacological treated animal groups was not significant. This finding suggested that the PPARγ-mediated transcription of apo-A-I was not different in treated groups and interestingly that a preserved PPARγ activity was also present in the LST group.

### PPARγ and the levels of the PPARγ-responsive gene SCARB1 were similar between treated groups

**Figure 3:**
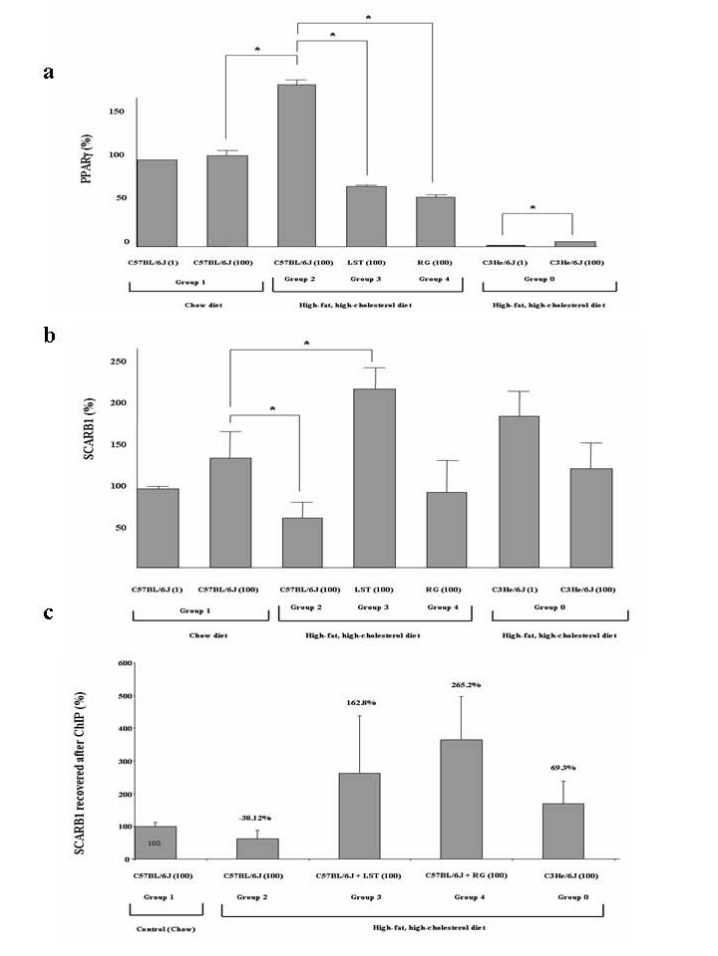
**Treatments preserve PPARγ activity and PPARγ responsive gene expression.** (**a**) PPARγ mRNA relative expression in pooled liver samples from analyzed groups normalized to GAPDH expression. Results are expressed as mean ± SEM percentage of mean values obtained from equal numbers of C57BL/6J and C3He/6J mice. *P < 0.05. (**b**). SCARB1 mRNA relative expression in liver samples from analyzed groups normalized to GAPDH expression. *P < 0.05 (**c**). ChIP with a PPARγ antibody of study groups was used to amplify a SCARB1 promoter fragment. ChIP figures percentages indicate increments or decrements regarding C57BL/6J mice fed HFHC diet. Data of C57BL/6J mice fed chow diet was established as 100%.

As seen in Figure 3a at day 100, a significant increase in PPARγ gene relative expression was observed in animals fed on HFHC diet with respect to chow (p = 0.01). No difference was observed in the PPARγ expression levels between LST and RG treated mice. Gene expression levels of the PPARγ response gene SCARB1 were next evaluated. At day 100 a significant decrease in SCARB1 gene expression in mice fed on HFHC diet with respect to mice fed on chow diet (p = 0.02) was observed. SCARB1 expression levels were significantly higher in hypercholesterolemic mice treated with LST in respect to untreated HFHC fed mice. Jointly considered PPARγ and SCARB1 expression data are suggestive of a preserved PPARγ activity in both pharmacological treated groups. To specifically evaluate this PPARγ preserved activity, ChIP assays were conducted. Following PPARγ-mediated immunoprecipitation assays revealed that there was a 38% reduction in the amplified level of SCARB1 promoter region that was accessible to PPARγ in mice fed with a HFHC diet compared to mice fed a control low-fat diet (p = 0.109) (Figure 3c). It seems that in mice fed on HFHC diet, in spite of a significant increase in the PPARγ expression levels, this nuclear receptor seems to be unable to efficiently bind to the PPRE site in the SCARB1 gene. In this context, we observed that in those animals fed on HFHC diet that were also treated with LST or RG, the amplified values of SCARB1 DNA were respectively 162% and 265% higher than those in C57BL/6J fed a HFHC diet. The difference between LST and RG groups did not reach statistical significance.

### Difference in CD36 relative gene expression between treated mice; rosiglitazone prevents Nrf2-dependent CD36 up-regulation

**Figure 4:**
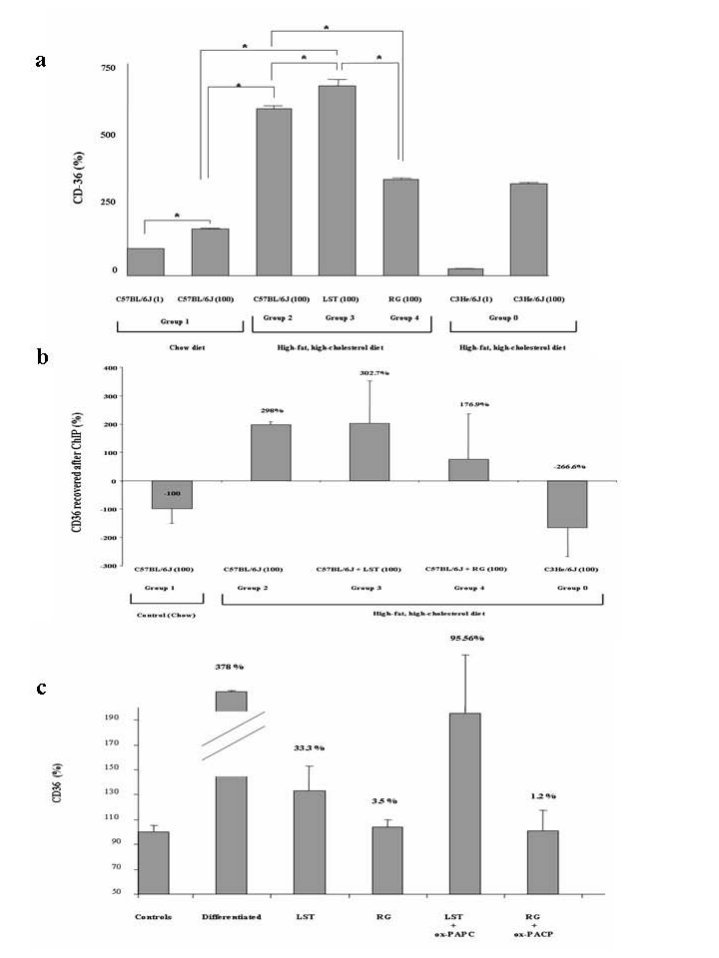
**Rosiglitazone prevents Nrf2-depedent CD-36 up-regulation in hypercholesterolemic C57BL/6J mice.** (**a**) CD36 mRNA relative expression in pooled liver samples from analyzed groups normalized to GAPDH expression. *P < 0.05. (**b**) ChIP with an Nrf2 antibody of study groups was used to amplify a CD36 promoter fragment. ChIP figures percentages indicate increments or decrements regarding C57BL/6J mice fed HFHC diet. Data of C57BL/6J mice fed chow diet was established as 100%. (**c**) Quiascent day-5 adipocytes incubated with RG and LST were treated with ox-PACP for additional 24 h. RNA was isolated to measure adipose protein 2 by conventional PCR and CD36 expression using real-time PCR. Values were normalized to GAPDH expression. Figure bar plots represent mean ± SEM percentage from vehicle-treated cells of at least 4 experiments with duplicate measurements.

Increased gene expression level of CD36 gene was shown in animals that were fed on HFHC diet with respect to chow-fed mice (p = 0.02) (Figure 4a). We observed a significant increase in CD36 relative expression level in mice treated with LST with respect to mice control groups feeding on either chow or HFHC diet (p < 0.05 for both). Mice that were fed on HFHC diet treated with RG showed higher but not significant CD36 relative gene expression level regarding chow-fed mice. A significant reduction in CD36 expression levels was observed in RG treated mice with regard to both HFHC control mice and HFHC mice treated with LST (p < 0.05 for both).

Previous results suggested similar levels of PPARγ activity between our treated groups. Studies have established the role of Nrf2 as a novel signalling pathway involved in the regulation of the CD36 gene. Evidence that ox-LDLs and certain oxidized lipids of LDL particles are inducers of Nrf2 [21] has been reported. Thus, we next explored the possibility that Nrf2 could be playing a pivotal role in the observed diminished total plasma LOOH levels in mice that fed a HFHC diet treated with RG with respect to either LST treated and untreated hypercholesterolemic mice as well as in the observed diminution in the relative expression levels of CD36 gene in the RG mice group with respect to the mice LST group. To address this possibility, ChIP assays using an antibody against Nrf2 followed with real-time analysis of the ARE site at the CD36 gene promoter identified as described in the methods were performed. A significant Nrf2-dependent increase in the amplified CD36 gene promoter region was observed in animals fed with a HFHC diet with respect to control chow mice (p < 0.05) (Figure 4b); similarly, mice treated with LST showed increased amplification levels of the CD36 promoter region regarding chow mice (p < 0.05 for both). Those RG treated mice showed moderate but significant lower amplification levels of the CD36 promoter fragment with respect to the amplification levels obtained in the control chow-fed group (Figure 4b). Remarkably, as depicted in Figure 4b, we found a 125.8% decrease in hypercholestelomic mice treated with RG with respect to LST treated mice (p = 0.02).

### Rosiglitazone prevents CD36 up-regulation in 3T3 differentiate cells exposed to ox-PACP

Adipocyte differentiated cells were treated with LST or RG at the doses and times previously described. Differentiation was confirmed by the increased oil-red staining and evaluating the relative expression of the adipogenic marker gene adipose protein 2 (ap2). Statistical comparisons revealed that cells treated with the differentiation cocktail showed significant higher level of ap2 amplified fragment regarding all analyzed groups. Those LST or RG treated cells showed similar levels of ap2 gene. Cells treated with both RG and LST showed diminished CD36 expression levels than those observed in differentiated untreated cells (p < 0.05 for both) (Figure 4c). However, no difference between both treated cell groups was observed. Then, we tried to simulate *in vivo *oxidative conditions by exposing treated cells to ox-PAPC. Cells treated with LST exposed to ox-PAPC showed no difference in the CD36 relative expression levels with respect to its differentiated cells group (p = 0.07) (Figure 4c). However, a statistical significant reduction in CD36 relative expression levels in cells treated with RG exposed to ox-PAPC with respect to differentiated control non-exposed cells (p = 0.03) was observed (Figure 4c).

Moreover, we found that the LOOH content in the supernatant of differentiated cells was significantly increased with respect to its undifferentiated controls (p = 0.007). This increase positively correlated with the CD36 gene expression levels (p = 0.034). On the contrary, we observed that both LST and RG treatments produced a significant decrease in the LOOH content with respect to differentiated cells group (p = 0.044 and p = 0.001, respectively). Again, no difference in LOOH levels was observed between treated cells. Cells treated with RG or LST exposed to ox-PACP showed mean lower levels of LOOH than those obtained in unexposed cells treated only with LST or RG (p < 0.001 for both). Statistical comparison showed no differences between either ox-PACP treated group. Notably, this occurred in spite of the fact that CD36 gene expression levels were observed to be higher in cells treated with LST plus ox-PACP regarding cells treated with RG plus ox-PACP.

### Assessing the relative expression of Nitric Oxide Synthase and Interleukin-6 genes

**Figure 5:**
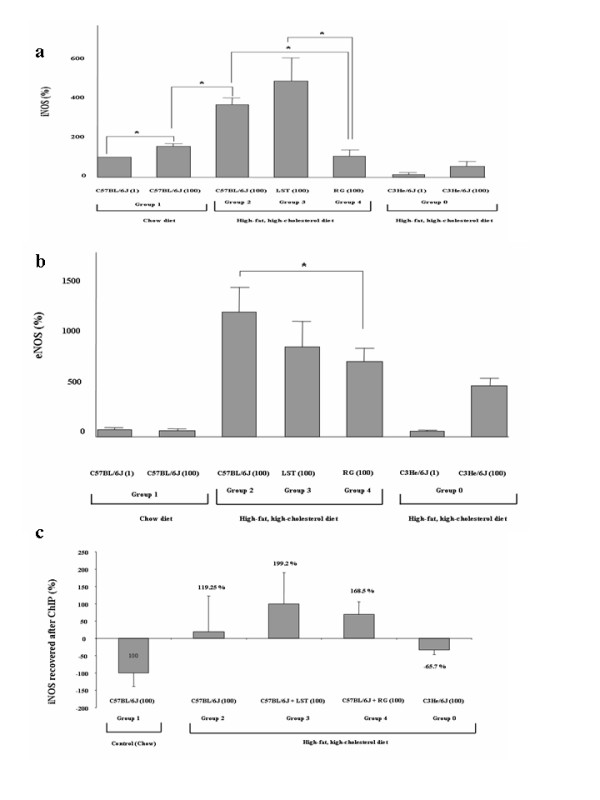
**Treatments modified iNOS gene expression while PPARγ-mediated iNOS amplification levels were similar.** (a) iNOS mRNA relative expression in pooled liver samples from analyzed groups normalized to GAPDH expression. *P < 0.05. (b) eNOS mRNA relative expression in pooled liver samples from analyzed groups normalized to GAPDH expression. Results are expressed as mean ± SEM percentage of mean values obtained from equal numbers of C57BL/6J and C3He/6J mice. *P < 0.05. (c) ChIP with a PPARγ antibody of study groups was used to amplify an iNOS promoter fragment. ChIP figures percentages indicate increments or decrements regarding C57BL/6J mice fed HFHC diet. Data of C57BL/6J mice fed chow diet was established as 100%.

RG treated mice showed a statistically significant reduction of the iNOS relative gene expression, with respect to both chow-fed animals and HHFC-fed mice treated with LST (p < 0.05 for both) (Figure 5a). IL-6 relative gene expression values although lowers in RG treated mice did not differ among analyzed groups (not shown).

Mean eNOS gene expression values in mice fed on a HFHC diet treated with RG or LST were smaller than those obtained in HFHC fed control mice. This reduction was significant for the comparison of mice treated with RG with respect to HFHC-fed control group (p = 0.04) (Figure 5b). Accordingly, group comparison showed that plasma nitric oxide metabolites values were found to be reduced in RG regarding LST treated mice (p = 0.02).

### Differences in the iNOS gene expression and its PPARγ dependence

ChIP assays using an antibody against PPARγ followed by real-time amplification of an iNOS gene fragment flanking the recently identified PPRE [43] at iNOS gene showed a 119% increase in average mean amplification values in mice under HFHC diet with respect to those obtained in chow-fed mice (-100%) (p = 0.1) (Figure 5c). In line with our previous results, amplification levels were higher after amplifying DNA from PPAR immunoprecipitates obtained from HFHC-fed mice treated with LST and RG (199% and 168% respectively). Comparison between treated groups yielded no difference (p > 0.05) (Figure 5c). Thus, although PPARγ expression levels were not increased in these groups regarding mice fed only on a HFHC diet, the ability to access PPRE binding sites in target genes seems to be preserved in both treated mice groups. Thus, the observed iNOS up-regulation in LST treated mice seems to be independent of the PPAR preserved activity.

## Discussion

In this study we demonstrated that the administration of RG or LST to diet-induced atherosclerosis susceptible mice fed a HFHC diet preserves to the same extent in both groups the activity of nuclear receptor PPARγ Conclusions derived from the analysis of the expression levels of PPARγ responsive gene SCARB1 and the amplification levels of PPRE binding sites of target genes amplified after ChIP which were shown to be approximately equal.

Previous studies in two murine models of atherosclerosis have shown that treatment with PPARγ agonists protected against the development of atherosclerosis [17-19]. However, lessons obtained from murine models of atherogenesis with or without diabetes should be viewed with caution given the known differences between vascular lesions from those animal models compared to those seen in humans [46]. Interestingly, we find that in spite of the similarly preserved PPARγ activity, RG administration was associated with a better "antiatherogenic" profile than this found in animals treated with LST exposed to the same diet. Thus, we found that hypercholesterolemic mice treated with RG showed inferior levels of total peroxidized lipids than those measured in hypercholesterolemic mice treated with LST. Moreover, when the relative gene expression levels of the iNOS and IL-6 genes were evaluated, we found lower expression levels in mice treated with RG with respect to those obtained in LST treated mice. Values reached significance for iNOS gene expression. Further enhancements of iNOS expression in mice that fed a HFHC diet treated with RG with respect to LST treated mice was not due to PPARγ-mediated transcriptional activity as deduced from ChIP assays.

Because there is a second target of PPARγ activation, the liver X receptor α whose activity was not analyzed in our work, care must taken with the interpretation of our results. Nevertheless, we suggested possible additional antioxidants and anti-inflammatory effects of the RG that seem to be partially independent of its action on PPARγ activity. It is important to note that in our experimental model, we also observed a difference in the HDL-cholesterol concentration level. Values were higher in mice treated with RG compared to those found in the LST treated group. Besides the key role of HDL particles in the regulation of cholesterol homeostasis by enhancement reverse cholesterol transport, it is known that HDL also exhibits antioxidant and anti-inflammatory properties that participate in its general antiatherogenic effect. The antioxidant ability of HDL is due to the apoprotein moiety and to the presence of associated enzymes [47]. The apoA-I has also been shown to be capable of removing seeding molecules from LDL, thus preventing the oxidation of LDL-derived phospholipids [38,48]. Therefore we considered if this observed HDL increase would justify by itself the best antioxidant profile obtained in mice treated with RG as compared to LST treated mice. We showed that the HDL particles isolated from both treated animal groups exhibited similar characteristics regarding their capacity to avoid the oxidation of PACPC by HpODE. However, these experiments do not exclude the fact that perhaps this difference in the HDL levels could be relevant by itself. The determinant role of apo A-I in increasing HDL levels has been clearly established in apoE-knockout mice [49]. According to this, it could be possible that non-evaluated differences in apo A-I concentrations, and therefore differences in HDL concentrations and activities, were justified by other transcriptional regulators that were not analyzed in our work. Interestingly, because of the existence of a peroxisome proliferator-responsive element (PPRE) at the apo A-I gene, it also could be possible that as suggested [18] the apo A-I gene might be positively regulated by PPARγ. This possibility was analyzed in our work. Thus, because we observed similar of PPARγ-mediated apo A-I gene transcription we concluded that the apo A-I is certainly positively regulated by PPARγ agonists but also indirectly we concluded that the PPARγ activity was preserved in both treated animal groups.

The role of PPARγ as a transcriptional regulator of CD36 gene expression has been previously established [9,17]. Surprisingly, we observed a statistical difference in the relative expression levels of the CD36 gene between both treated animal groups while no difference was observed in the relative expression levels of PPARγ-responsive gene SCARB1. Our results suggested differences at inflammatory and oxidative levels between both treated animal groups being less harmful in mice treated with RG than in mice treated with LST. Studies have demonstrated PPARγ induction in human monocytes following exposure to oxLDL [9,50]. Notably new evidence has been provided that Nrf2 plays an essential role in regulating CD36 expression [18,21]. Moreover, ox-LDLs are known to activate Nrf2-mediated gene responses [21]. Therefore, we thought that in our experimental model some similar situation would justify the observed difference in the CD36 gene relative expression levels where Nrf2 could be important. Thus, we performed gene expression analysis and ChIP experiments and concluded that in HFHC-fed mice treated with RG the smaller expression levels of the hepatic CD36 gene were observed as opposed to HFHC-fed mice treated with LST because RG prevents Nrf2-mediated CD36 up-regulation. It has been reported that PPARγ ligands, 15-deoxy-Delta(12,14)-prostaglandin J(2) (PGJ(2)), and troglitazone inhibits Nrf2-induced expression of the tromboxane synthase gene in macrophages [51]. Because Nrf2 also regulates CD36 gene expression, authors [21] suggested that the transcriptional regulators PPARγ and Nrf2 may interact functionally to modulate CD36 gene expression. As has been previously noted, we demonstrated similarly preserved PPARγ activity in hypercholesterolemic mice treated with RG or LST. Therefore we deduced that RG seems to afford some additional antiaterogenic properties that prevent Nrf2-depedent CD-36 up-regulation which are partially independent of its metabolizing properties as a PPARγ agonist.

We did not find statistically significant differences in the eNOS relative gene expression levels between treated groups with similarly preserved PPARγ activity. Authors suggest that TZDs protective effects on vascular wall cells are mediated by its ability to up-regulate eNOS gene expression [52,53]. This effect of TZDs may explain its *in vivo *vasodilating and blood pressure-lowering effects and, at least for some TZDs, seems to be independent of their PPARγ metabolizing effects. However, although the molecular mechanisms by which TZDs increase NO release are not completely worked out, they are likely to depend on each particular TZD. For example Troglitazone, has an alpha-tocopherol (vitamin E) moiety that is not present in other TZDs, and treatment of hepatocytes with vitamin E and Troglitazone but not with RG led to an inhibition of phosphoenolpyruvate carboxykinase gene expression suggesting a PPARγ-independent, antioxidant-related mechanism [54]. Goya et al [52] suggested that the antioxidant and anti-inflammatory properties observed following troglitazone administration to cells were linked to the 6-hydroxychromonanes structure of troglitazone. Jiang et al [55] proved that γ-tocopherol diminished prostaglandin E2 (PGE2) synthesis in macrophages. Therefore, some TZDs share common anti-inflammatory properties with PPARγ independency, which are partially mediated by its structural relations with tocols. Gonon et al [56] recently provide evidences that RG results in protection against myocardial contractile dysfunction induced by ischemia reperfusion in mice receiving RG by mediating eNOs protein phosphorilation without increasing total eNOS concentration.

Our work has concentrated in analyzing the hepatic expression of PPARγ and Nrf2 target genes and the valuation of some serum markers. Although it is important to go deep in the in vivo situation it is also important to recognize the differences of regulation of the expression of genes regulated by PPAR and Nrf2 are tissue [21] and context-dependent [57]. Thus, caution is required in the interpretation of our results. Particularly, further in vivo studies should be necessary to explore the role of Nrf2 transcription factor in modulating the progression of foam cell formation and atherosclerosis.

## Conclusion and Clinical Perspective

The present study indicates that the administration of RG or LST to diet-induced atherosclerosis susceptible mice fed a HFHC diet preserves in both groups the PPARγ activity. RG seems to have antioxidant effects in addition to its main metabolic activity in a manner that actually diminishes rather than increases the expression of the gene CD36 by inhibiting its Nrf-2-dependent up-regulation. Despite the favourable effects of RG observed in this study, it must be acknowledged that the meta-analysis of Nissen et al [58] on RG and cardiovascular risk raises the hypothesis that TZDs treatments may increase the risk of myocardial infarction and cardiovascular events. However, the general consensus is that this hypothesis needs further evaluation [59]. Currently, animal and human studies suggest that PPARγ ligands have anti-inflammatory, antioxidant and vasculo-protective protective effects. However, though there are similarities in TZD-induced improvement in insulin sensitivity between experimental models and humans, there are also substantial differences. Studies have focused on the role of adipocytokines in metabolic control and their regulation by TZDs. Nevertheless, growing evidence suggests that the vasculo-protective effects of PPARγ ligands can be dissociated from its metabolizing actions. Thus, there may be important implications for the development of improved anti-atherogenic and antidiabetic drug treatments. Additionally, the identification of potential benefits associated with the common molecular structure among these drugs will have interesting clinical repercussions.

## Abbreviations

AT_1_R = Angiotensin Type 1 Receptor; ChIP = Chromatin immunoprecipitation; eNOS (NOS3) = Nitric Oxide Synthase 3; HDL = High Density Lipoprotein; HFHC = high-fat high-cholesterol; IL-6 = Interleukin 6; iNOs (NOS2) = Nitric Oxide Synthase, inducible; LOOH = Lipid Hydroperoxide; LST = Losartan; NO = Nitric Oxide; Nrf2 = Nuclear Factor Erythroid 2-Like 2; PPARγ = Peroxisome Proliferator-Activated Receptor-Gamma; RG = Rosiglitazone.
